# The Neurobiological Impact of Ghrelin Suppression after Oesophagectomy

**DOI:** 10.3390/ijms18010035

**Published:** 2016-12-26

**Authors:** Conor F. Murphy, Carel W. le Roux

**Affiliations:** 1 Diabetes Complications Research Centre, Conway Institute, University College Dublin, Dublin 4, Ireland; cmurph1010@gmail.com; 2 Gastrosurgical Laboratory, Sahlgrenska Academy, University of Gothenburg, 40530 Gothenburg, Sweden

**Keywords:** ghrelin, ghrelin suppression, GHS-R1A, oesophageal carcinoma, appetite, anticipatory feeding, reward-induced feeding, oesophagectomy

## Abstract

Ghrelin, discovered in 1999, is a 28-amino-acid hormone, best recognized as a stimulator of growth hormone secretion, but with pleiotropic functions in the area of energy homeostasis, such as appetite stimulation and energy expenditure regulation. As the intrinsic ligand of the growth hormone secretagogue receptor (GHS-R), ghrelin appears to have a broad array of effects, but its primary role is still an area of debate. Produced mainly from oxyntic glands in the stomach, but with a multitude of extra-metabolic roles, ghrelin is implicated in complex neurobiological processes. Comprehensive studies within the areas of obesity and metabolic surgery have clarified the mechanism of these operations. As a stimulator of growth hormone (GH), and an apparent inducer of positive energy balance, other areas of interest include its impact on carcinogenesis and tumour proliferation and its role in the cancer cachexia syndrome. This has led several authors to study the hormone in the cancer setting. Ghrelin levels are acutely reduced following an oesophagectomy, a primary treatment modality for oesophageal cancer. We sought to investigate the nature of this postoperative ghrelin suppression, and its neurobiological implications.

## 1. Introduction

Kojima et al. ascertained, in 1999, that the endogenous ligand for GHS-R1a was ghrelin, a peptide hormone capable of stimulating the anterior pituitary gland to secrete growth hormone [1]. Ghrelin is produced by cells within the gastric fundus, known as Gr-cells. This discrete population of enteroendocrine cells is a subtype of oxyntic or X/A-like cells, and represents approximately 20% of this cell population [2]. The majority of circulating ghrelin is in the form of desacyl ghrelin, but ghrelin-*O*-acyl-transferase (GOAT) facilitates the post-translational addition of an acyl side-chain to pro-ghrelin at position 3 of the serine residue-forming acyl ghrelin. Ghrelin octanoylation by GOAT allows it to bind GHS-R1a, and thus is key to its orexigenic and metabolic activity [3]. Other preproghrelin gene-derived peptides include obestatin. The year following its discovery, Tschop et al. demonstrated that ghrelin targeted areas of the brain to assist in the regulation of body weight, glucose metabolism and food intake [4]. Subsequent discoveries have implicated it in a multitude of functional roles executed via diverse but complementary mechanisms, with central and peripheral interactions. The definitive part ghrelin plays in everyday neurobiology is incompletely understood. Research in the area of ghrelin suppression in post-upper gastrointestinal surgery, although limited, has aided in the attempt to answer some questions regarding its role.

## 2. Discussion

### 2.1. Energy Homeostasis

Ghrelin’s role in energy homeostasis appears to be mediated by action upon hypothalamic circuits [5,6]. GHS-R is created in afferent neurons of the vagus, then axonally transported to be expressed mainly in the gastric mucosa [7]. Once bound by ghrelin, an orexigenic effect is stimulated. GHS-R dampens activity within the afferent neuron, signalling to the nucleus tractus solitarius, which transmits the stimulus to neuropeptide Y (NPY)- and agouti-related peptide (AgRP)-containing neurons within the hypothalamus [8,9]. NPY/AgRP–double-knock-out mice are resistant to ghrelin′s orexigenic stimulus, and inhibition of this system, both pharmacologically and immunologically, serves to block ghrelin-induced feeding effects, highlighting the importance of this hypothalamic pathway [2]. The arcuate nucleus is an important target for ghrelin when it comes to regulating food intake [10,11], but other hypothalamic regions can also lead to the promotion of positive energy balance when stimulated with ghrelin administration, such as the paraventricular nucleus (PVN) [12,13], the dorsomedial hypothalamus (DMH) [14] and the lateral hypothalamus (LHA) [15]. The hippocampus has been implicated in the behavioural aspect of feeding [16,17], with evidence of widespread expression of GHS-R in hippocampal neurons [18]. Activation of ventral hippocampal neurons by ghrelin increases meal frequency and size in murine models [19]. This effect is potentially mediated via direct communication between ghrelin-activated hippocampal neurons and neurons in the LHA that express orexin, a neuropeptide [20].

Ghrelin was found to have many more effects, both peripherally and centrally, such as energy conservation, by reducing energy expenditure via suppression of brown adipose tissue (BAT) thermogenesis [21,22,23,24,25] and policing glucose metabolism, and by preventing muscular atrophy through promotion of skeletal muscle cell fusion [26,27]. Ghrelin stimulates the secretion of both gastric acid and motilin [28,29], and also enhances vasodilation and cardiac contractility [30,31,32,33]. Influencing motility via these peptides may perhaps play a part in its role in anticipatory feeding and in increasing meal size. Although initially thought to be an inert degradation product of acylated ghrelin, there is evidence suggesting that desacyl ghrelin is an active hormone that can both agonise and antagonise acyl ghrelin, as well as having its own receptor [34]. Murine studies have demonstrated that it may have anorexigenic activity via mechanisms such as a reduced gastric emptying rate [35].

### 2.2. Hunger Hormone?

Ghrelin’s well-described orexigenic effect on food intake is regulated by sensing the presence of nutrients, and relaying this information to the brain. As such, ghrelin is frequently thought of as the “hunger hormone” [6,36,37,38,39], but this is a one-dimensional view of what is clearly a multifunctional hormone. It has also, perhaps more accurately, been described as a meal-anticipatory hormone based on the fact that its levels rise prior to anticipated feeding, independent of energy deprivation levels [38,40], with rapid suppression of circulating levels post-prandially. Furthermore, ghrelin receptor–null mice, under meal entrainment feeding circumstances, do not demonstrate the same response [41]. This association with conditioned appetite and feeding, which appears to be mediated through a neural conversation between the ventral hippocampus and the lateral hypothalamus, may link a number of the demonstrated neurobiological effects of ghrelin, although this area is incompletely understood. Nonetheless, it is intimately linked with hunger levels [37,42], and binds to receptors located within subcortical areas involved in satiety and food intake regulation [9,10,11,43].

### 2.3. Neurobiological Role of Ghrelin

Both ghrelin and its receptor are widely expressed in multiple regions of the brain [6,44,45,46,47], many of which are associated with feeding behaviour [48]. While having a pivotal role in energy homeostasis as an orexigenic hormone, research over the past two decades has also implicated ghrelin in a myriad of neurophysiological functions, such as learning and memory [19,49,50], psychological stress, mood, anxiety [51,52], depression [53,54,55] and addiction [56]. Further central effects include influencing the circadian rhythm [57,58,59], modulation of anxiety and stress [53,60,61], and stimulating reward-seeking behaviour, as well as the motivation to eat [62,63,64,65,66,67]. Using functional magnetic resonance imaging (fMRI), Malik et al. demonstrated increased activation in areas such as the amygdala and orbitofrontal cortex during intravenous ghrelin infusion and exposure to pictures of food [68]. At the level of the ventral tegmental area (VTA), but not the nucleus accumbens (NAc), ghrelin increases motivation for food, reflected by an increased lever-pressing behaviour for sucrose pellets in a progressive ratio task [69]. As evidenced by these findings, as well as its discussed hippocampal effects, ghrelin appears to recruit pathways involved in reward-based eating behaviour, as opposed to those associated with energy homeostasis. This extra-homeostatic feeding impulse, in addition to ghrelin’s anticipatory feeding role, may represent alternate functions, which, when altered post-operatively, can potentially exhibit abnormal physical and psychological sequelae.

Interestingly, quality of life scores are reduced following oesophagectomy, with functional scores in areas such as emotional issues and anxiety lower than average [70]. Overall, the impact of prolonged ghrelin suppression on these vital processes is not well known. Whether it may be partly accountable for post-operative neuropsychological symptoms is an area yet to be explored. As a natural stimulator of growth hormone (GH), and also GH-releasing hormone and somatostatin release, ghrelin acts as a communicator between the pituitary gland, and the gastrointestinal tract. This signaling pathway facilitates the regulation of anabolism and substrate use, matching them to available energy resources. Ghrelin can stimulate more efficient energy usage via hypothalamic pathways in times of nutrient deficit, in tandem with its aforementioned suppressive effect on BAT thermogenesis [4]. Disruption of these effects post-operatively may lead to inappropriate regulation of energy expenditure. Overall, its link with appetite, energy homeostasis and energy expenditure has led to extensive research within the areas of obesity and metabolic surgery, as well as the cancer cachexia syndrome.

### 2.4. Post-Operative Ghrelin Suppression

The incidence of oesophageal adenocarcinoma has been rising steadily, correlating with the rise in metabolic disease and obesity [71,72,73]. In keeping with this, oncological outcomes have improved, in patients treated with curative intent, due to factors such as increased centralisation of surgery, improved staging and peri-operative care and well-structured screening programmes [74]. For patients with locally advanced cancer receiving multimodal therapy, the recent neoadjuvant chemoradiotherapy (CROSS) trial quoted three-year survival and five-year survival rates as 58% and 47%, respectively [75,76,77]. With this improved survivorship has come a renewed focus on health-related quality of life post-operatively. Amongst the frequently reported post-operative complications are weight loss, loss of appetite and early satiety. Approximately one in three patients who are disease-free have over 15% weight loss at three years [78]. Calorie malnutrition is thus a major issue.

Upper gastrointestinal surgery, such as Roux-en-Y gastric bypass (RYGB), also leads to changes in body weight and appetite, and is often associated with a reduction in ghrelin levels [79,80,81,82]. Subsequent data, however, show that ghrelin levels frequently return to pre-operative levels within the first year after this surgery in humans [83] and within six weeks after surgery in mice [84], with some studies showing an increase in ghrelin post-RYGB [85]. Additionally, compared to wild-type control mice, in ghrelin–knock-out mice, vertical sleeve gastrectomy is equally efficient in lowering body weight [86], which would suggest a ghrelin-independent effect in this type of bariatric surgery. The mechanism of this ghrelin suppression is an area of much research. One potential factor is supraphysiological increases in post-prandial gut hormones, which arise from the small bowel, as observed in specific post-surgical states [87,88,89], including bariatric and oesophagogastric cancer surgery. GLP-1 may exhibit this effect via its insulinotropic action, as insulin is a known modulator of plasma ghrelin and was capable of suppressing it by 19%–64% in one study [90]. Although long-term data are not available describing the trend of this phenomenon over time, perhaps this mechanism could account for the relative recovery of ghrelin levels in the longer-term. Doki et al. hypothesized that the level of ghrelin circulating after such an operation and the amount of residual stomach may be directly proportional. Indeed, they reported that following oesophagectomy and gastrectomy, ghrelin levels were reduced by approximately 10% to 50%, respectively, while colectomy did not lead to any change [80]. Furthermore, in a study comparing gastroduodenal (GD) with gastrojejunal (GJ) reconstruction following partial gastrectomy for gastric cancer, ghrelin levels fell dramatically in both groups immediately post-operatively, but at one year the levels in GD patients increased more distinctly. This suggests that the duodenum may have a crucial compensatory ghrelin-producing role [91]. A less explored area of interest is the body mass index-(BMI)-independent change in gut microbiota composition following bariatric surgery [92], given that this “virtual endocrine organ” may influence gut hormone secretion [93].

Miyazaki et al. demonstrated an association between reduced BMI and reduced plasma ghrelin levels at six to 24 months post-oesophagectomy with gastric tube reconstruction, with ghrelin levels falling from 130.8 ± 13 fmol/mL pre-operatively to 50.6 ± 5.9 and 73.2 ± 9.7 fmol/mL at seven days and a mean of 20.1 months (range, six to 23.5 months) post-operatively. However, ghrelin levels returned to pre-operative levels (146 ± 44.8 fmol/mL) over 36 months (mean 53.4, range 39–80 months) post-operatively in this study, while BMI continued to decrease. The authors concluded that the reason for this medium-term discordance was unclear, likely multi-factorial and would need further investigation. Weight loss and plasma ghrelin have also not been found to correlate perennially in other studies [79,94]. Nevertheless, a phase II randomised trial looking at the clinical effects of a 10-day course of exogenous ghrelin administration post-oesophagectomy commencing on the first day of oral food intake concluded that it improved post-operative oral intake and attenuated weight loss in the acute period, and therefore warranted further evaluation to assess medium- and long-term effects [95]. Furthermore, persistently elevated ghrelin levels were noted in patients with diet-induced weight loss, which often tends to be unsustainable [82,96]. Overall, these results could implicate ghrelin as a contributor to the mechanism underlying the weight loss in some cases, as well as potentially playing a role in the altered quality of life scores demonstrated following these operative procedures. However, it is unlikely that ghrelin is the major contributor to either early, or late, post-operative weight loss. Other factors, such as early satiety induced by exaggerated post-prandial gut hormone responses, are probably implicated, particularly in longer-term weight loss. Although these data are mainly from non-oesophageal cancer cohorts, the results imply that ghrelin has, at best, a small role in weight loss following upper gastrointestinal cancer surgery.

### 2.5. Ghrelin and Cancer Cachexia

The cancer cachexia-anorexia syndrome is a multifactorial process commonly seen in cancer, involving skeletal muscle and adipose tissue atrophy [97,98]. The issue of weight loss is thus exaggerated in oesophageal cancer patients, who subsequently undergo oesophagectomy, predisposing them to further weight loss [79,94]. There are many components to this syndrome, such as a pro-inflammatory response, and physical symptoms of the tumour such as dysphagia [99]. While circulating levels of ghrelin have been shown to be elevated in cancer cachexia, this did not correlate with an increase in appetite, suggesting it was a compensatory response to weight loss [100]. This is consistent with its role in the prevention of muscular atrophy. Ghrelin has been studied in the management of cachexia in murine models [101], due to its ability to release growth hormone, regulate appetite, increase gastric emptying, and its anti-inflammatory properties [5]. Anamorelin, a synthetic GSH-R agonist, had a favourable clinical response profile in advanced cancer patients over 12 weeks of treatment [102]. It has also been effective in increasing food intake and body weight, as well as performance status, in cachexia associated with chronic obstructive pulmonary disease (COPD) [103], and in improving muscle wasting by increasing muscle strength in chronic heart failure-(CHF)-associated cachexia [31]. Ghrelin is a potential targetable factor in the prevention of weight loss and other adverse outcomes associated with cancer, while post-operative suppression of ghrelin may adversely affect patient outcome. All in all, while there have been some promising developments and short-term results, there are no long-term reported effects resulting from the therapeutic modulation of ghrelin, nor its incidental post-operative suppression.

## 3. Conclusions

Ghrelin has a multitude of effects, especially within the area of energy homeostasis, appetite regulation, meal initiation, as well as in anticipatory feeding behaviours. It exhibits these actions via complex neurobiological pathways. Although weight loss is inconsistently correlated with ghrelin levels in these post-operative patients, its ties to such powerful neural avenues indicate that it may at least have a role in influencing weight fluctuation through multiple mechanisms. It is clear that acylated ghrelin, and perhaps desacyl ghrelin, have a central role in the hormonal interplay between the gastrointestinal tract and the central nervous system, which does suggest that suppression following upper gastrointestinal operations is likely to have a metabolic effect at both sites. Its intimacy with appetite, a powerful and evocative sensation, lends weight to the concept that ghrelin has an essential role in influencing energy consumption, but the inconsistency of this effect in the post-operative setting and the unexplained mechanism renders its role unclear as of yet.

In conclusion, this fascinating hormone provides an array of stimulating hypotheses and certainly warrants future further investigation with the hope of harnessing the potential of this metabolic player to ultimately improve patient quality of life. However, while the impact of ghrelin suppression with potential anorexigenic sequelae has been examined in the context of oesophageal carcinoma and subsequent surgical interventions, neurobiological consequences of persistently low levels have not been widely explored.
